# Percutaneous Pedicle Screw for Thoracolumbar Fractures: A Long-term Follow-up

**DOI:** 10.1055/s-0044-1779701

**Published:** 2024-03-21

**Authors:** Marcos Vinícius da Rocha Furtado, Gabriel Santos Braga, Roberto Rossanez, Carlos Fernando Pereira da Silva Herrero

**Affiliations:** 1 Faculdade de Medicina de Ribeirão Preto, Universidade de São Paulo, Ribeirão Preto, São Paulo, Brasil

**Keywords:** minimally invasive surgical procedures, spinal fractures, patient outcome assessment

## Abstract

**Objective:** This is a retrospective cohort study to analyze the long-term outcomes of thoracolumbar spine fracture patients who underwent minimally invasive percutaneous fixation.

**Methods:** The cases of 17 patients with thoracolumbar spine fractures who had percutaneous fixation between 2009 and 2011 were the subject of a retrospective analysis. Clinical and radiographic variables were collected. For the clinical evaluation the questionnaires SF-36 and Oswestry were used. Radiographic parameters were evaluated using fracture's classification based on Magerls's criteria, the fractured vertebra's wedging angle, and the affected segment's segmental Cobb angle. The measures were made at different stages: before surgery, immediately after surgery, one year later, and at a late follow-up (5 years later). Trauma associated injuries, post-surgical and implant related complications were among the additional information taken into account.

**Results:** The SF-36 questionnaire showed averages above 63,5% in all domains in the late postoperative data (from 5 years after the surgery). Oswestry questionnaire answers showed minimal or no physical limitations in 80% of the patients with a mean score of 10,8%±10,5. The average preoperative Cobb angle value was 5,53° ± 13,80° of kyphosis, the immediate postoperative 2,18° ± 13,38° of kyphosis, one year postoperative 5,26 ± 13,95° of kyphosis, and the late follow-up 8,78° ± 15,06° of kyphosis. The mean correction was 3,35°, and mean loss of correction was 6,6°. There were no complications observed, no case of neurological deficit, infection or implant failure occurred.

**Conclusion:** Thoracolumbar vertebrae fractures can be surgically treated with positive late clinical and radiological outcomes and low complication rates using a minimally invasive percutaneous method.

## Introduction

Spine fractures and the associated injuries are related to high-energy trauma and determine low return-to-work rates compared to injuries to other organs and systems. 1 It may be associated with chronic pain, 2 function limitation of correlated systems such as the respiratory, sequelae deformities, permanent mobility loss, fatigue, and neurological injuries. 3 When located in the thoracic and lumbar spines, neurological injuries occur in up to 30% of cases and constitute an important cause of limitations and disabilities. 2

Traumatic spine fractures often affect the thoracolumbar transition, a transition area between the rigid thoracic and flexible lumbar segments, becoming the most significant biomechanical stress area. 4 North American data show approximately 160.000 cases of these fractures annually. 4

Treatment options include the non-surgical approach with rest and use of orthosis; and the surgical approach that promotes immediate stabilization, potential decompression, and deformity correction. 5 The conservative treatment presents a potential complication of neurological deficit associated with the immobility required in this treatment in up to 20% of cases. 6 Conventional open surgery has potential complications such as blood loss, infections, postoperative pain, and paravertebral muscle atrophy. 6 7 8

Surgical treatment of thoracic and lumbar spine fractures is necessary in cases of biomechanical instability, compression related neurological deficit, lesions that contraindicate non-surgical treatment. 9 Nevertheless, treatment algorithms and criteria for instability, fragmentation of the vertebral body, and the need for arthrodesis associated with fixation are targets of controversy in the literature. 8 10 11

The percutaneous surgical approach emerged as an option and an attempt to reduce complications associated with open surgery and decrease surgical time and hospital stay length. 6 12 Therefore, questions such as the exposure to radiation, graft placement, and consolidation evolution, in addition to the absence of direct neurological decompression, remain unknown. 2 12 Thus, the objective of the present study is to evaluate the late results of percutaneous fixation surgery in patients with thoracolumbar spine fractures.

## Materials and Methods

This study was approved by the Ethics Committee on Human Research under the number of the Certificate of Release (CAAE- 42660015.9.0000.5440).

A retrospective cohort study of database was conducted, patients diagnosed with thoracolumbar fractures submitted to surgical treatment using a minimally invasive percutaneous fixation method were evaluated. These procedures were conducted at a Spine Surgery reference service between 2009 and 2011. Cases of tumoral pathological fractures, osteometabolic and infectious diseases were excluded from the selection.

The patients included in the study were operated on by the same surgeon, with Sextant® system implants (Medtronic Sofamor Danek USA, Inc. Memphis, TN, USA) using technique described in the preliminary results of this study. 8 Patient mobilization was allowed according to postoperative pain and limitation due to associated injuries.

Clinical and radiographic data were collected. The clinical data of interest were age, sex, trauma mechanism and presence of associated injuries, postoperative complications, and late follow-up of the quality of life and functional capacity SF-36 13 and Oswestry Disability Score -ODI. 14

The radiographic data obtained was classified by the following parameters: Magerl et al. 15 criteria, measurement of the fractured vertebra wedging angles, segmental Cobb angle, 16 and radiographic documentation of complications such as implants related (loosening or breakages). These criteria were availed at different stages, preoperative period, immediate postoperative period, one-year postoperative period, and late postoperative (more than five years after surgery). All parameters were measured by a spine surgeon with more than ten years of experience, using the same software (Osirix) and the same computer.

The data obtained were organized in spreadsheets, and data analysis was performed using the Excel® program, with the results presented as a percentage.

## Results

Sixteen men (94.12%) and one woman (5.88%) were enrolled in the study. Age at the time of trauma ranged between 16 and 60 years old (29.1 ± 12.28). Sixteen patients (94.12%) were treated by fixing one vertebra above and one below the fractured level. One patient (5.88%) with concomitant T11 and T12 fractures underwent fixation of the affected vertebrae. One patient (5.88%) underwent an additional anterior approach for decompression and arthrodesis.

Regarding the trauma mechanism, motorcycle accidents accounted for 11 cases (64.70%), while falls from height accounted for 6 cases (35.30%). Regarding the affected level, 12 patients (70.6%) had thoracolumbar transition (T11 to L2) affected and 5 (29.40%) the lumbar region (L3 to L5). Type A (subdivision 2 and 3) fractures were diagnosed eight times (35.29%), type B five (29.41%), and type C was diagnosed in other six fractures (35.29%), according to Magerl et al. 15 classification. Associated injuries were present in 9 patients (52.94%). Only one patient had a neurological deficit (Magerl A.3) in the preoperative period and underwent a different approach via the anterior approach with complete recovery after surgery. No infection was identified, and there was no need for reoperation.

The postoperative follow-up of the patients occurred between 5 and 9 years. There was a sample loss of 7 patients, six did not respond to the call, and one death occurred unrelated to the fracture treatment. In the analysis of the SF-36 13 questionnaires, a variation of 50% to 100% (0.89 ± 0.19%) was observed for social aspects, between 45% and 100% (0.80 ± 0.19%) for functional capacity, 42% to 95% (0.64 ± 0.20) for the assessment of general health, 45% to 100% (0.80 ± 0.19) for functional limitations, from 48% to 88% (0.72 ± 0.14) for mental health and between 30% and 95% (0.69 ± 0.20) for vitality ( Table 1 ).

**Table 1:** Score of patients on the SF-36 Questionnaire

Patient	Functional capacity	Physical Limitation	Pain	General Health Status	Vitality	Social aspects	Emotional Limitations	Mental health
1	100%	100%	100%	80%	80%	100%	100%	88%
2	95%	75%	72%	72%	80%	100%	100%	88%
3	100%	100%	100%	75%	65%	100%	100%	68%
4	80%	100%	62%	47%	80%	100%	100%	68%
5	85%	100%	72%	95%	95%	100%	100%	72%
6	85%	100%	90%	80%	80%	100%	100%	88%
7	45%	0%	62%	42%	60%	50%	0%	52%
8	55%	0%	41%	52%	40%	75%	100%	64%
9	95%	60%	71%	59%	75%	100%	100%	80%
10	60%	0%	20%	42%	30%	63%	100%	48%

**Fig. 1:**
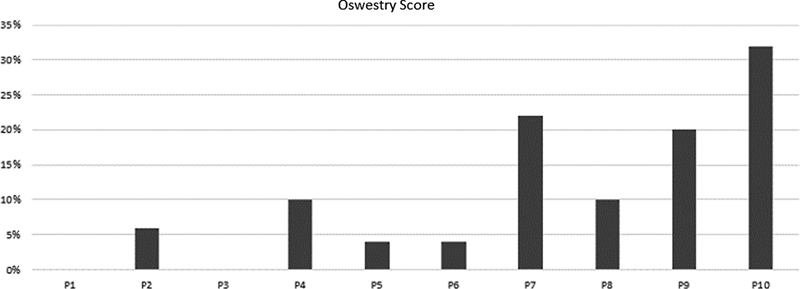
Patientńs scores on the Oswestry Questionnaire.

**Fig. 2:**
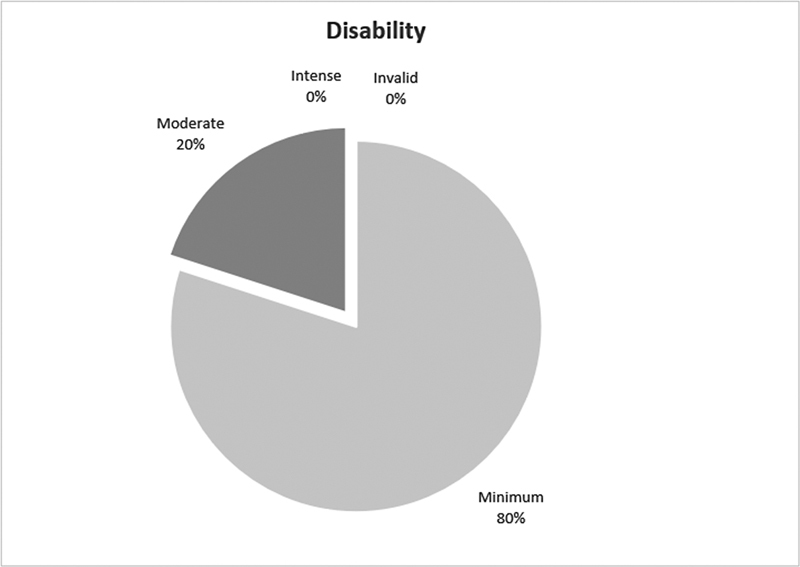
Data interpretation.

Oswestry questionnaire 14 results evidenced a mean score of 10.8% ± 10.5 associated to low back pain, while eight patients (80%) reported minimal disability or absence of disability ( Figs. 1 and 2 ).

**Fig. 3:**
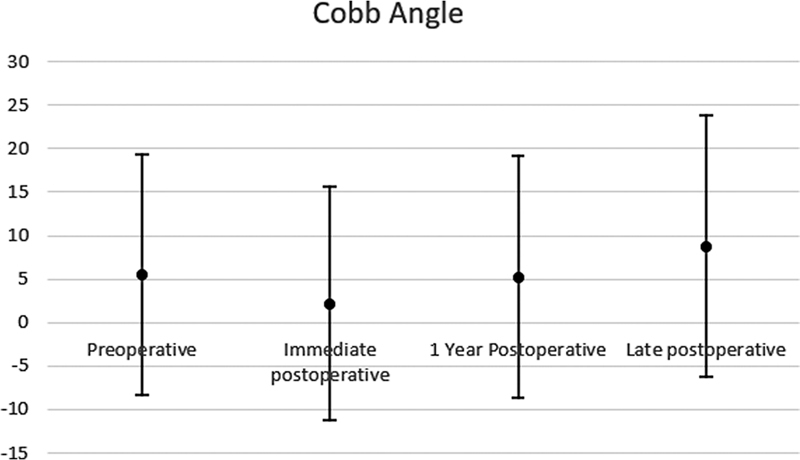
Mean Cobb angles per period.

Radiographic parameters revealed a vertebral wedging that ranged from 0° to 29° of kyphosis (mean of 13.06° ± 7.55°) in preoperative period. The preoperative Cobb angle ranged from 28° of kyphosis to 22° of lordosis (5.53° ± 13.80° of kyphosis), immediate postoperative from 27° of kyphosis to 20° of lordosis (2.18° ± 13, 38° of kyphosis), one-year postoperative period from 30° of kyphosis to 24° of lordosis (5.26° ± 13.95° of kyphosis) and late follow-up (from 5 years) from 37° of kyphosis to 12°, 7° of lordosis (8.78° ± 15.06° of kyphosis) ( Fig. 3 ).

**Fig. 4:**
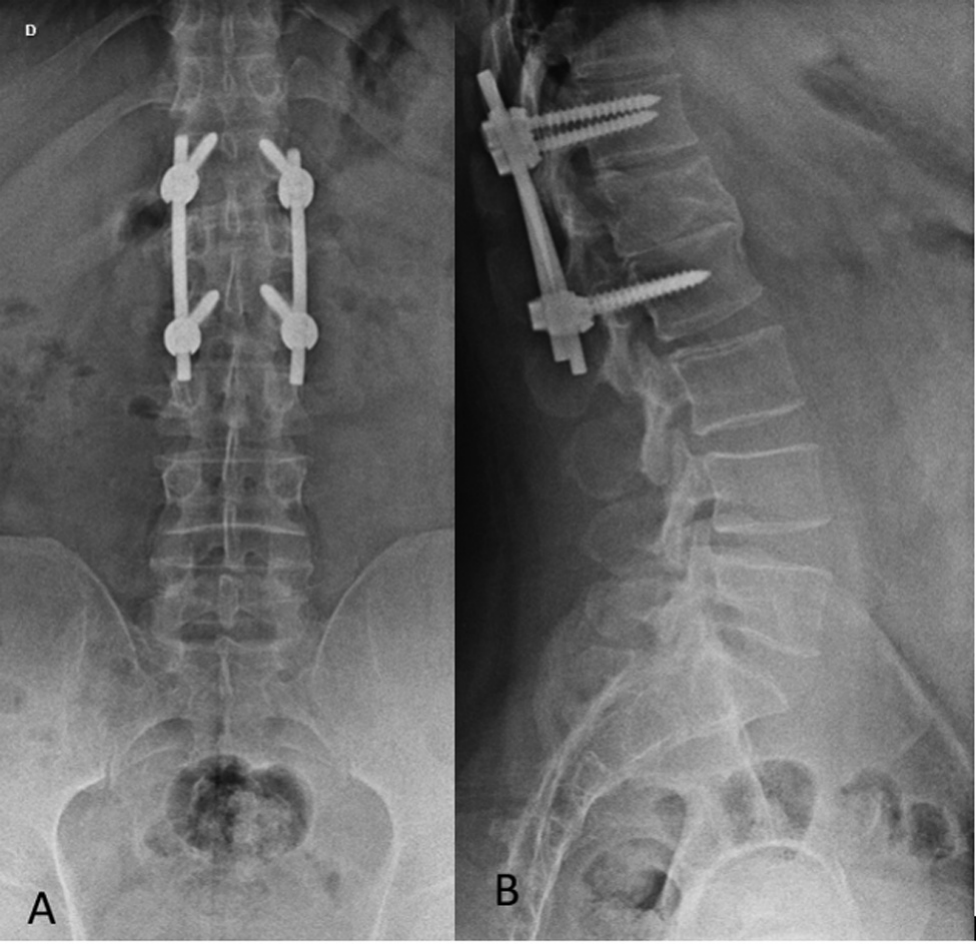
Immediate post-operative radiograph of a patient undergoing minimally invasive percutaneous fixation in anteroposterior (A) and lateral (P) views.

**Fig. 5:**
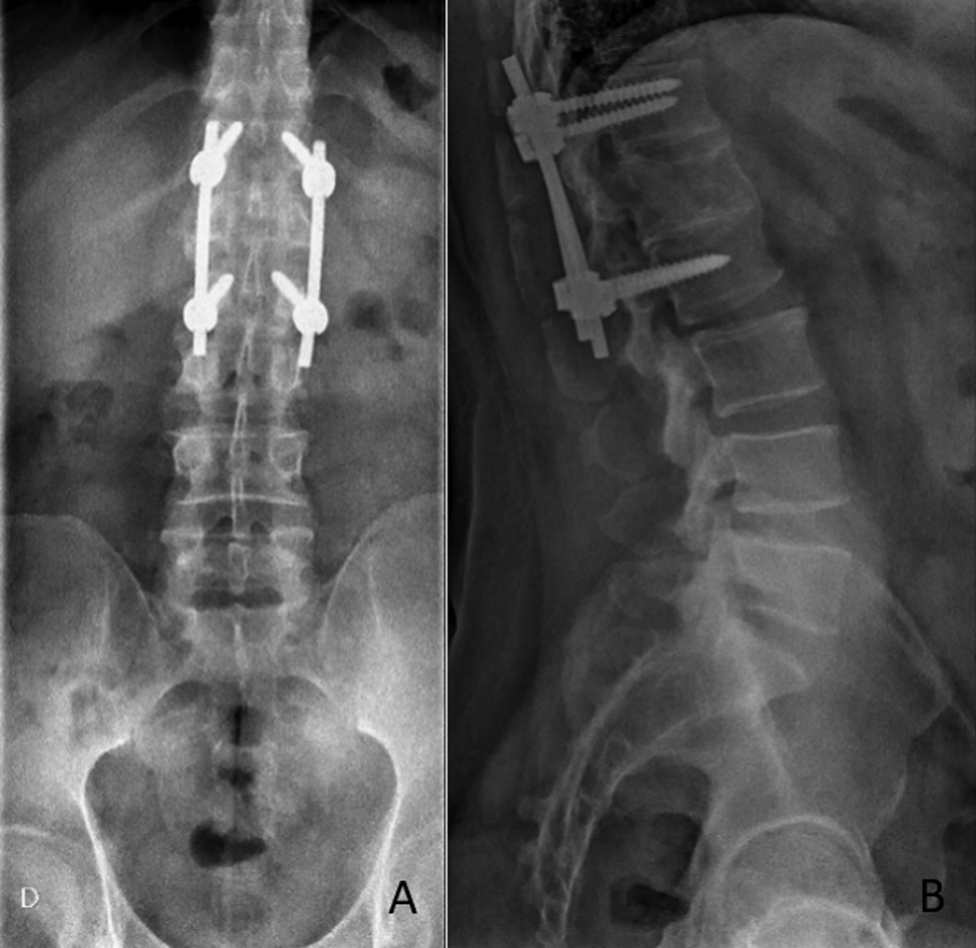
Late follow-up radiograph of the same patient in Fig. 4 . Performed 6 years after the procedure, in anteroposterior (A) and lateral (P) views.

The mean correction was 3.35°, using preoperative and immediate postoperative data. Between the immediate postoperative and late follow-up the mean correction loss was 6.6°. Despite the degree loss of correction, no signs of implant loosening or failure were observed in late follow-up as osteolysis ( Figs. 4 and 5 ).

## Discussion

Although there has yet to be a consensus in the literature on the best approaches for treating thoracolumbar fractures of the spine, 11 alternatives for reducing complications related to each method are discussed. Nonsurgical treatment may require prolonged physical restriction, which often impacts work and financial life, and may lead to the need for bed restriction and its complications, such as neurological deterioration. Other challenges involved are the intricate control of consequent deformities and the patient's lack of understanding and cooperation. 17 18

The minimally invasive approach to the treatment of thoracolumbar fractures of the spine emerges as an alternative surgical treatment with a lower degree of aggression and denervation of the paravertebral muscles, in addition to other complications of conventional open surgery, such as increased intramuscular pressure, ischemia, bleeding and greater risk of infection. 11 19 20 21

Previous studies managed to show lower rates of complications in this modality. 19 22 23 Objectively, reduction in postoperative pain, intraoperative blood loss, surgery time, hospital stay, and drainage of the surgical site have already been described. 6 However, the minimally invasive approach presents questions, for example, regarding radiation exposure, the lower possibility of graft placement and consequent risks in the evolution of consolidation, in addition to the possibility of direct neurological decompression 2 6 that require direct decompression of fragments in the medullary canal. 2 These particularities can always be resolved by completing a minimally invasive open decompression. 12

In the present study, no form of fixation was sufficient to maintain the correction of kyphosis obtained during the initial surgical procedure over the long term, a frequent finding in other studies that address postoperative follow-up. 16 22 24 25 26 27 On average, the return of kyphosis to baseline values was found one year after the procedure. The use of polyaxial pedicle screws can explain this finding. 19 Studies comparing polyaxial and monoaxial screws (with no movement at the body-head interface and promoting more substantial leverage) have shown less loss of correction in individuals treated for thoracolumbar fractures with monoaxial screw systems. 28 Nevertheless, we considered the minimum loss of correction associated to the 9 years of follow up and no clinical symptoms an explanation for the consolidation assurance.

Other questions about the loss of kyphosis correction relate to the probable lower rate of arthrodesis consolidation in patients treated with percutaneous fixation. Nevertheless, other studies have already evaluated the use of arthrodesis in the fixation of thoracolumbar fractures, concluding that there is no significant difference in clinical and radiological aspects. 10

Regarding the improvement in quality of life, studies that used the Visual Analog Pain Scale and ODI in the minimally invasive treatment of spinal fractures showed better results from this modality compared to conventional open surgery. 29

Kumar et al. 30 (2015) applied the ODI in patients undergoing conservative treatment, minimally invasive surgery, and open surgery. A mean score of 32 (with a range between 12 and 46) was found in those treated with conservative and 14 (with a range between 4 and 26) in those treated with open surgery, contrasting with 4 (ranging between 0 and 10) for minimally invasive surgery at the 18-month and 30-month follow-up. Then, a significant difference favored the less invasive approach 18. In the present study, similar data were found, confirmed by the improvement of the ODI and SF-36 indices in the studied patients. When present at late follow-up, disability was reported to be moderate in about 20% of patients.

Although the data in the literature used for comparison in the late follow-up refer to ODI questionnaires applied within 30 months after the chosen treatment. 30 This study had the analysis carried out more than five years after the operation. A much lower score was observed in the minimally invasive approach, which can result from a much less aggressive procedure and does not imply absence from activities for as long a time as in a conservative approach.

As a limitation, this study maintained late follow-up up to a maximum of 9 years. In addition, it brings results from a small and specific sample of patients affected by fractures, young adults affected by higher-energy trauma, failing to obtain data with the other extreme, elderly patients with bone thinning and susceptible to fractures even with lower-energy trauma. On the other hand, the absence of postoperative complications and the performance of the procedures by the same surgeon allow for good reliability in the analysis of the results obtained. Besides, we believe that the percutaneous vertebral fixation is a straightforward technique once the spine surgeon follows the technical description correctly.

## Conclusion

The minimally invasive percutaneous fixation can be used as an option for the treatment of thoracolumbar fractures. It showed satisfactory outcomes, including clinical and radiographic parameters, with low complication rates in the studied sample.
