# Supplementary material for: Percutaneous Pedicle Screw for Thoracolumbar Fractures: A Long-term Follow-up
Source: Rev Bras Ortop (Sao Paulo). 2024 Mar 21;59(1):e101–6. doi: 10.1055/s-0044-1779701 (PMC10957267; doi:10.1055/s-0044-1779701)
Supplement: Related texts

# Research article: Parafuso pedicular percutâneo em fraturas toracolombares: Acompanhamento em longo prazo

**Keywords:** fraturas da coluna vertebral, procedimentos cirúrgicos minimamente invasivos, avaliação de resultados da assistência ao paciente

## Abstract

**Objetivo:** Este é um estudo de coorte retrospectivo para analisar os desfechos em longo prazo de pacientes com fratura da coluna toracolombar submetidos à fixação percutânea minimamente invasiva.

**Métodos:** Os casos de 17 pacientes com fraturas da coluna toracolombar submetidos à fixação percutânea entre 2009 e 2011 foram objeto de análise retrospectiva. Variáveis clínicas e radiográficas foram coletadas. A avaliação clínica foi baseada nos questionários SF-36 e Oswestry. Os parâmetros radiográficos foram avaliados de acordo com a classificação de fratura baseada nos critérios de Magerls, o ângulo de cunha da vértebra fraturada e o ângulo de Cobb do segmento acometido. As medidas foram feitas em diferentes momentos: antes da cirurgia, imediatamente após a cirurgia, um ano depois e no acompanhamento tardio (cinco anos depois). Lesões associadas a traumas, complicações pós-cirúrgicas e relacionadas a implantes também foram consideradas.

**Resultados:** O questionário SF-36 apresentou médias acima de 63,5% em todos os domínios no período pós-operatório tardio (a partir de cinco anos após a cirurgia). As respostas do questionário Oswestry mostraram limitações físicas mínimas ou nulas em 80% dos pacientes, com pontuação média de 10,8% ± 10,5%. O valor médio do ângulo de Cobb foi de 5,53° ± 13,80° de cifose no período pré-operatório, 2,18° ± 13,38° de cifose no pós-operatório imediato, 5,26 ± 13,95° de cifose no pós-operatório de um ano e de 8,78° ± 15,06° de cifose no período pós-operatório tardio. A correção média foi de 3,35° e a perda média de correção foi de 6,6°. Não foram observadas complicações, casos de déficit neurológico, infecções ou falhas do implante.

**Conclusão:** As fraturas das vértebras toracolombares podem ser tratadas cirurgicamente com desfechos clínicos e radiológicos tardios positivos e baixas taxas de complicações usando um método percutâneo minimamente invasivo.

## Introdução

As fraturas da coluna vertebral e as lesões associadas estão relacionadas a traumas de alta energia e determinam baixas taxas de retorno ao trabalho em comparação a lesões em outros órgãos e sistemas. 1 Podem estar associadas à dor crônica, 2 limitação funcional de sistemas correlacionados, como o sistema respiratório, sequelas de deformidades, perda permanente de mobilidade, fadiga e lesões neurológicas. 3 Na coluna torácica e lombar, as lesões neurológicas ocorrem em até 30% dos casos e constituem uma importante causa de limitações e incapacidades. 2

As fraturas traumáticas da coluna tendem a acometer a transição toracolombar, uma área entre o segmento torácico, que é rígido, e o segmento lombar, que é flexível. Assim, esta é a área de estresse biomecânico mais significativa. 4 Dados norte-americanos revelam cerca de 160.000 casos dessas fraturas ao ano. 4

Dentre as opções terapêuticas, estão a abordagem não cirúrgica com repouso e uso de órtese e a abordagem cirúrgica, que promove estabilização imediata, possível descompressão e correção de deformidades. 5 Uma possível complicação do tratamento conservador é o déficit neurológico associado à imobilidade exigida em até 20% dos casos. 6 A cirurgia convencional aberta apresenta possíveis complicações como perda de sangue, infecções, dor pós-operatória e atrofia muscular paravertebral. 6 7 8

O tratamento cirúrgico das fraturas da coluna torácica e lombar é necessário em casos de instabilidade biomecânica, déficit neurológico relacionado à compressão e lesões que contraindicam o tratamento não cirúrgico. 9 No entanto, a literatura é controversa quanto aos algoritmos de tratamento e aos critérios de instabilidade, fragmentação do corpo vertebral e necessidade de artrodese em associação à fixação. 8 10 11

A abordagem cirúrgica percutânea surgiu como uma opção na tentativa de reduzir as complicações associadas à cirurgia aberta e diminuir o tempo cirúrgico e o tempo de internação hospitalar. 6 12 Porém, questões como exposição à radiação, colocação do enxerto e evolução da consolidação, além de a ausência de descompressão neurológica direta, ainda não foram elucidadas. 2 12 Assim, o objetivo deste estudo é avaliar os desfechos tardios da cirurgia de fixação percutânea em pacientes com fraturas da coluna toracolombar.

## Materiais e Métodos

Este estudo foi aprovado pelo Comitê de Ética em Pesquisa com Seres Humanos sob número de Certificado de Liberação (CAAE- 42660015.9.0000.5440).

O estudo de coorte retrospectiva de banco de dados avaliou pacientes com diagnóstico de fratura toracolombar submetidos a tratamento cirúrgico com método de fixação percutânea minimamente invasiva. Esses procedimentos foram realizados em serviço de referência em Cirurgia da Coluna entre 2009 e 2011. Casos de fraturas patológicas tumorais, doenças osteometabólicas e infecciosas foram excluídos da amostra.

Os pacientes incluídos no estudo foram operados pelo mesmo cirurgião e receberam implantes do sistema Sextant® (Medtronic Sofamor Danek USA, Inc. Memphis, TN, EUA) de acordo com a técnica descrita nos resultados preliminares desta pesquisa. 8 A mobilização do paciente foi permitida conforme a dor pós-operatória e a limitação decorrente das lesões associadas.

Dados clínicos e radiográficos foram coletados. Os dados clínicos de interesse foram idade, sexo, mecanismo de trauma e presença de lesões associadas, complicações pós-operatórias e acompanhamento tardio da qualidade de vida e capacidade funcional conforme os questionários SF-36 13 e *Oswestry Disability Score* (ODI). 14

Os dados radiográficos obtidos foram classificados pelos seguintes parâmetros: critérios de Magerl et al., 15 medida dos ângulos de cunha das vértebras fraturadas, ângulo de Cobb segmentar 16 e documentação radiográfica de complicações, como implantes relacionados (soltos ou fraturados). Esses critérios foram utilizados em diferentes períodos, incluindo pré-operatório, pós-operatório imediato, pós-operatório de um ano e pós-operatório tardio (mais de cinco anos após a cirurgia). Todos os parâmetros foram medidos por um cirurgião de coluna com mais de dez anos de experiência utilizando o mesmo software (Osirix) e o mesmo computador.

Os dados obtidos foram organizados em planilhas e a análise dos dados foi realizada no programa Excel®, com os resultados apresentados em porcentagem.

## Resultados

Dezesseis homens (94,12%) e uma mulher (5,88%) foram incluídos no estudo. A idade no momento do trauma variou entre 16 e 60 anos (29,1 ± 12,28). Dezesseis pacientes (94,12%) foram tratados com fixação de uma vértebra acima e outra abaixo do nível fraturado. Um paciente (5,88%) com fratura concomitante de T11 e T12 foi submetido à fixação das vértebras acometidas. Um paciente (5,88%) foi submetido a uma abordagem anterior adicional para descompressão e artrodese.

Quanto ao mecanismo de trauma, os acidentes motociclísticos foram responsáveis por 11 casos (64,70%), enquanto as quedas de altura representaram seis casos (35,30%). O nível acometido foi a transição toracolombar (T11 a L2) em 12 pacientes (70,6%) e a região lombar (L3 a L5) em cinco pacientes (29,40%). Houve oito (35,29%) fraturas do tipo A (subdivisões 2 e 3), cinco (29,41%) do tipo B e seis (35,29%) do tipo C de acordo com a classificação de Magerl et al. 15 Lesões associadas foram observadas em nove pacientes (52,94%). Apenas um paciente apresentou déficit neurológico (Magerl A.3) no pré-operatório e foi submetido a uma abordagem diferenciada pela via anterior com recuperação completa após a cirurgia. Nenhuma infecção foi identificada e não houve necessidade de reoperação.

O acompanhamento pós-operatório dos pacientes compreendeu um período entre cinco e nove anos. Houve perda amostral de sete pacientes, sendo que seis não atenderam ao chamado e um foi a óbito por motivo não relacionado ao tratamento da fratura. Na análise dos questionários SF-36 13 , observou-se variação de 50% a 100% (0,89 ± 0,19%) em aspectos sociais, 45% a 100% (0,80 ± 0,19%) na capacidade funcional, 42% a 95% (0,64 ± 0,20) na avaliação do estado geral de saúde, 45% a 100% (0,80 ± 0,19) em limitações funcionais, 48% a 88% (0,72 ± 0,14) em saúde mental e 30% e 95% (0,69 ± 0,20) em vitalidade ( Tabela 1 ).

**Tabela 1:** Escore dos pacientes no Questionário SF-36

Paciente	Capacidade funcional	Limitação física	Dor	Estado geral de saúde	Vitalidade	Aspectos sociais	Limitações emocionais	Saúde mental
1	100%	100%	100%	80%	80%	100%	100%	88%
2	95%	75%	72%	72%	80%	100%	100%	88%
3	100%	100%	100%	75%	65%	100%	100%	68%
4	80%	100%	62%	47%	80%	100%	100%	68%
5	85%	100%	72%	95%	95%	100%	100%	72%
6	85%	100%	90%	80%	80%	100%	100%	88%
7	45%	0%	62%	42%	60%	50%	0%	52%
8	55%	0%	41%	52%	40%	75%	100%	64%
9	95%	60%	71%	59%	75%	100%	100%	80%
10	60%	0%	20%	42%	30%	63%	100%	48%

A pontuação média no questionário Oswestry 14 foi de 10,8% ± 10,5 associada à dor lombar. Oito pacientes (80%) relataram incapacidade mínima ou ausência de incapacidade ( Figs. 1 e 2 ).

**Fig. 1:** Escores dos pacientes de acordo com o Questionário Oswestry.

**Fig. 2:** Interpretação dos dados.

Os parâmetros radiográficos revelaram um acunhamento vertebral entre 0° a 29° de cifose (média de 13,06° ± 7,55°) no pré-operatório. O ângulo de Cobb variou de 28° de cifose a 22° de lordose (5,53° ± 13,80° de cifose) no pré-operatório, 27° de cifose a 20° de lordose (2,18° ± 13,38° de cifose) no pós-operatório imediato, 30° de cifose a 24° de lordose (5,26° ± 13,95° de cifose) um ano após a cirurgia e 37° de cifose a 12,7° de lordose (8,78° ± 15,06° de cifose) no acompanhamento tardio (a partir de cinco anos) ( Fig. 3 ).

**Fig. 3:** Valores médios dos ângulos de Cobb por período.

A correção média foi de 3,35° segundo os dados pré-operatórios e pós-operatórios imediatos. Entre o pós-operatório imediato e o acompanhamento tardio, a perda média de correção foi de 6,6°. Apesar da perda do grau de correção, não foram observados sinais de afrouxamento ou falha do implante no acompanhamento tardio, como osteólise ( Figs. 4 e 5 ).

**Fig. 4:** Radiografia no período pós-operatório imediato de um paciente submetido à fixação percutânea minimamente invasiva em projeção anteroposterior (A) e em perfil (B).

**Fig. 5:** Radiografia de acompanhamento tardio do mesmo paciente mostrado na Fig. 4 , realizada seis anos após o procedimento, em projeção anteroposterior (A) e em perfil (B).

## Discussão

Embora ainda não haja consenso na literatura sobre as melhores abordagens para o tratamento das fraturas toracolombares da coluna vertebral, 11 são discutidas alternativas para redução das complicações relacionadas a cada método. O tratamento não cirúrgico pode exigir restrição física prolongada, o que muitas vezes impacta a vida profissional e financeira, podendo levar à necessidade de restrição ao leito e suas complicações, como a deterioração neurológica. Outros desafios são o intrincado controle das deformidades consequentes e a falta de compreensão e cooperação do paciente. 17 18

A abordagem minimamente invasiva para o tratamento das fraturas toracolombares da coluna vertebral surge como alternativa de tratamento cirúrgico com menor grau de agressão e denervação dos músculos paravertebrais, além de redução das complicações da cirurgia aberta convencional, como aumento da pressão intramuscular, isquemia, sangramento e maior risco de infecção. 11 19 20 21

Estudos anteriores demonstraram menores taxas de complicações com esta modalidade. 19 22 23 Objetivamente, já foram descritas redução da dor pós-operatória, da perda sanguínea intraoperatória, do tempo de cirurgia, do período de internação e da drenagem do sítio cirúrgico. 6 Porém, a abordagem minimamente invasiva é questionada, por exemplo, quanto à exposição à radiação, à menor possibilidade de colocação de enxerto e os consequentes riscos na evolução da consolidação, além da possibilidade de descompressão neurológica direta, 2 6 que requer descompressão direta de fragmentos no canal medular. 2 Estas particularidades podem sempre ser resolvidas por meio de uma descompressão aberta minimamente invasiva. 12

Neste estudo, nenhuma forma de fixação foi suficiente para manutenção em longo prazo da correção da cifose obtida no primeiro procedimento cirúrgico, um achado frequente em outros estudos que abordam o acompanhamento pós-operatório. 16 22 24 25 26 27 O retorno da cifose aos valores basais foi constatado em média um ano após o procedimento. O uso de parafusos pediculares poliaxiais pode explicar esse achado. 19 Estudos comparando parafusos poliaxiais e monoaxiais (sem movimento na interface corpo-cabeça e promovendo alavancagem mais substancial) mostraram menor perda de correção em indivíduos com fraturas toracolombares tratados com sistemas de parafusos monoaxiais. 28 No entanto, consideramos que a perda mínima de correção associada aos nove anos de acompanhamento e a ausência de sintomas clínicos indicam a consolidação adequada.

Outras dúvidas sobre a perda da correção da cifose dizem respeito à provável menor taxa de consolidação da artrodese em pacientes submetidos à fixação percutânea. Apesar disso, outros estudos já avaliaram o uso da artrodese na fixação das fraturas toracolombares, concluindo que não há diferença significativa em aspectos clínicos e radiológicos. 10

Em relação à melhora na qualidade de vida, estudos que utilizaram a Escala Visual Analóga de Dor e o ODI após o tratamento minimamente invasivo de fraturas da coluna vertebral mostraram desfechos melhores dessa modalidade em comparação à cirurgia aberta convencional. 29

Kumar et al. 30 aplicaram o ODI em pacientes submetidos a tratamento conservador, cirurgia minimamente invasiva e cirurgia aberta. A pontuação média foi 32 (com variação entre 12 e 46) nos tratados com cirurgia conservadora e 14 (com variação entre 4 e 26) naqueles tratados com cirurgia aberta, contrastando com a pontuação de 4 (variando entre 0 e 10) obtida após a cirurgia minimamente invasiva nos acompanhamentos de 18 e 30 meses. Assim, uma diferença significativa favoreceu a abordagem menos invasiva 18 . O presente estudo encontrou dados semelhantes, confirmados pela melhora dos índices ODI e SF-36. Quando presente no acompanhamento tardio, a incapacidade foi relatada como moderada por cerca de 20% dos pacientes.

Embora os dados de literatura utilizados para comparação no acompanhamento tardio se refiram a ODIs aplicados até 30 meses após o tratamento escolhido, 30 este estudo analisou dados obtidos mais de cinco anos após a cirurgia. A pontuação foi bem menor na abordagem minimamente invasiva, o que pode ser decorrente da menor agressividade do procedimento e na ausência de afastamento das atividades por tanto tempo como na abordagem conservadora.

Como limitação deste estudo, o acompanhamento tardio ocorreu até o período máximo de nove anos. Além disso, os resultados se referem a uma amostra pequena e específica de pacientes acometidos por fraturas, adultos jovens que sofreram traumas de maior energia, e não temos dados do outro extremo, ou seja, pacientes idosos com adelgaçamento ósseo e suscetíveis a fraturas mesmo por traumas de menor energia. Por outro lado, a ausência de complicações pós-operatórias e a realização dos procedimentos pelo mesmo cirurgião permitem boa confiabilidade na análise dos resultados obtidos. Também acreditamos que a fixação vertebral percutânea é uma técnica simples desde que o cirurgião de coluna siga corretamente a descrição técnica.

## Conclusão

A fixação percutânea minimamente invasiva pode ser uma opção no tratamento das fraturas toracolombares. Seus desfechos foram satisfatórios, inclusive em parâmetros clínicos e radiográficos, com baixas taxas de complicações na amostra estudada.
